# Cold argon-oxygen plasma species oxidize and disintegrate capsid protein of feline calicivirus

**DOI:** 10.1371/journal.pone.0194618

**Published:** 2018-03-22

**Authors:** Hamada A. Aboubakr, Sunil K. Mor, LeeAnn Higgins, Anibal Armien, Mohammed M. Youssef, Peter J. Bruggeman, Sagar M. Goyal

**Affiliations:** 1 Department of Veterinary Population Medicine and Veterinary Diagnostic Laboratory, College of Veterinary Medicine, University of Minnesota, St. Paul, MN, United States of America; 2 Food Science and Technology Department, Faculty of Agriculture, Alexandria University, Aflaton St, El-Shatby, Alexandria, Egypt; 3 Department of Biochemistry, Molecular Biology, and Biophysics & Proteomics Center for Mass Spectrometry, University of Minnesota, St. Paul, MN, United States of America; 4 Department of Mechanical Engineering, College of Science and Engineering, University of Minnesota, Minneapolis, MN, United States of America; Consejo Superior de Investigaciones Cientificas, SPAIN

## Abstract

Possible mechanisms that lead to inactivation of feline calicivirus (FCV) by cold atmospheric-pressure plasma (CAP) generated in 99% argon-1% O_2_ admixture were studied. We evaluated the impact of CAP exposure on the FCV viral capsid protein and RNA employing several cultural, molecular, proteomic and morphologic characteristics techniques. In the case of long exposure (2 min) to CAP, the reactive species of CAP strongly oxidized the major domains of the viral capsid protein (VP1) leading to disintegration of a majority of viral capsids. In the case of short exposure (15 s), some of the virus particles retained their capsid structure undamaged but failed to infect the host cells *in vitro*. In the latter virus particles, CAP exposure led to the oxidation of specific amino acids located in functional peptide residues in the P2 subdomain of the protrusion (P) domain, the dimeric interface region of VP1 dimers, and the movable hinge region linking the S and P domains. These regions of the capsid are known to play an essential role in the attachment and entry of the virus to the host cell. These observations suggest that the oxidative effect of CAP species inactivates the virus by hindering virus attachment and entry into the host cell. Furthermore, we found that the oxidative impact of plasma species led to oxidation and damage of viral RNA once it becomes unpacked due to capsid destruction. The latter effect most likely plays a secondary role in virus inactivation since the intact FCV genome is infectious even after damage to the capsid.

## Introduction

Cold atmospheric-pressure gaseous plasma (CAP) is one of the most promising non-thermal technologies that has gained attention recently due to its strong bactericidal activity [1–7]. The CAP is defined as a partially ionized gas consisting of photons, ions, free electrons, and reactive species including radicals [OH^•^, ^•^NO, O_2_ (a^1^Δ_g_)], molecules (O_3,_ H_2_O_2_, HNO_2_), and atoms (O^•^, N^•^) [8]. Although the anti-bacterial effects of CAP have been investigated extensively, only a few studies have dealt with its antiviral activity [9–15]. In a previous investigation [16], we reported strong *in vitro* virucidal activity (> 6 log_10_ TCID_50_/0.1 ml) against feline calicivirus (FCV) by 15s exposure of virus suspension to the effluent of a radio frequency (RF)-driven CAP jet with gas flow of 99% argon and 1% oxygen operated in an open-air environment. A combination of detailed scavenger measurements combined with positive controls allowed us to conclude that both singlet oxygen and O_3_ are the key species inactivating FCV in water [17].

The effects of plasma species, particularly singlet oxygen, on the essential functionalities of the virus (e.g., host recognition and binding with the host receptors, entry into the host cell, and genome replication) are unknown. However, Yasuda et al. [18] reported results, which suggest that the inactivation of bacteriophage lambda by cold plasma was mainly due to oxidation of the coat protein, and to a lesser extent, by damage to the DNA. A similar mode of action of CAP on bacteriophage MS2 [19] and human adenovirus [20], has been suggested but Sakudo et al. [14] found that the reactive chemical species of nitrogen plasma inactivated an adenovirus by damaging its genomic DNA without any alteration in its capsid protein, hexon, or penton [14]. Similarly, ultraviolet light inactivates viruses by damaging their nucleic acids [21].

Singlet oxygen produced by photosensitization has been found to impair the replication of bacteriophage MS2 by destroying its genome and by oxidizing several peptides in the viral assembly proteins. The oxidation effect on capsid proteins was very small and subsequently the host binding and injection of nucleic acid were mildly affected [22]. However, the inactivation of bacteriophage F2 by ozone treatment was attributed to the breakdown of viral coat proteins, which hindered the adsorption of the phage to the host cells [23].

Although some insights are available on the possible impact of plasma species on viruses, a clear understanding of the process is lacking partially because different combinations of plasma sources and virus models have been used and different explanations have been provided on the mode of virus inactivation. The purpose of this study was to determine the biological impact of plasma-produced reactive oxygen and nitrogen species (RONS) that leads to inactivation of FCV. A clear understanding of the loss in essential virus functionalities due to plasma exposure in liquid would potentially help in optimizing the exposure conditions for maximum viral inactivation effect. We used several approaches to achieve the goals of this study. (i) the effect on virus morphology and capsid proteins was studied by the use of transmission electron microscopy (TEM) and quantitative reverse transcription-polymerase chain reaction coupled with ethidium monoazide (EMA-RT-qPCR), (ii) mass spectrometry and quantitative proteomics were used to study the oxidative effects of plasma produced species on viral capsid proteins, and (iii) quantitative RT-PCR (RT-qPCR) and genome integration were used to study the possible effects on viral RNA.

## Materials and methods

### Plasma generation setup

**Fig 1:**
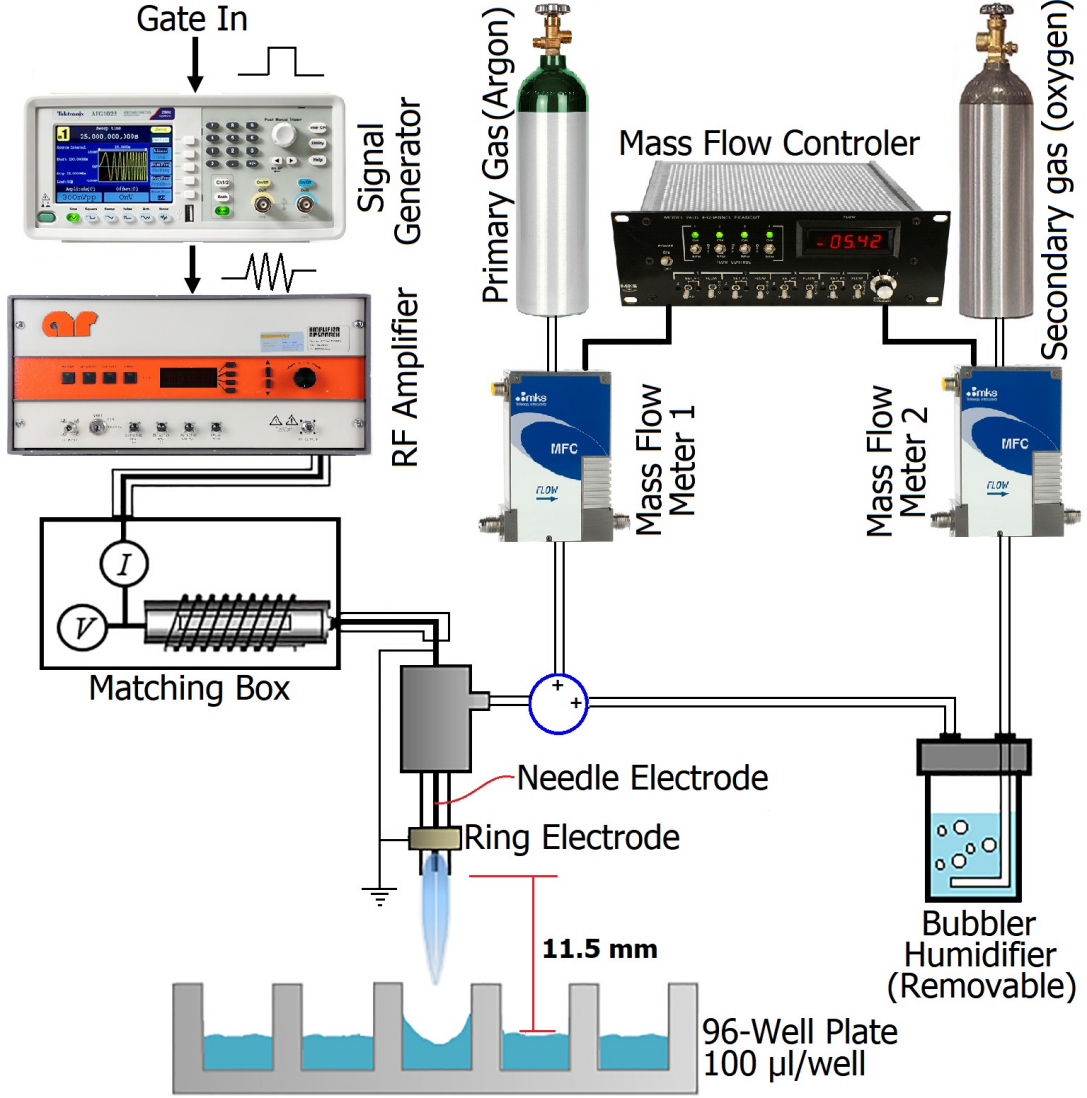
Schematic diagram of the plasma jet including sample treatment and electrical and gas inputs.

The plasma jet used in this study (Fig 1) was described by van Gils et al., [3]. Briefly, plasma is ignited in Ar which is blown through a 1.9 mm quartz tube at 1.5 standard liters per minute (slm) by radio frequency (RF) power (13.56 MHz) modulated at a frequency of 20 kHz with a duty cycle of 20%. This study was done at a time-averaged plasma dissipated power of 2.5 W measured as described in Hofmann et al. [24] using 99%Ar+1%O_2_ as the feed gas. The amount of gas admixture was controlled by using mass flow controllers (MKS GE50). The plasma was operated in the air environment of the laboratory causing the presence of small amounts of air in the plasma jet effluent, which is the source of reactive nitrogen species generated in this Ar-O_2_ plasma (CAP) jet as demonstrated in our previous study [17]. For virus inactivation experiments, we placed a sterile 96-well microtiter plate (containing 100 μl aliquots per well of FCV suspended in sterilized Millipore water) below the plasma jet at 11.25 mm exposure distance (as measured from plasma quartz tube nozzle to the surface of the virus suspension) (see Fig 1). After 15 s. and 2 min exposure times, the samples were removed followed by titration of treated and non-treated (control) FCV samples as described below. Because of the evaporation effect of the gas flow, we observed a decrease in the volume of the exposed samples and hence the volumes of exposed samples were readjusted to 100 μl by adding compensating volumes of distilled water (DW) into samples before titration.

### Virus propagation and purification

Strain 255 of FCV was used as a model for non-enveloped RNA viruses. The FCV has also been used as a cultivable surrogate for HuNoV in many studies [25–27]. The virus was grown and titrated in Crandell-Reese feline kidney (CRFK) cells which was obtained from the Veterinary Diagnostic Laboratory, University of Minnesota. The CRFK cells were grown in minimum essential medium (MEM) with Earle's Salts and L-Glutamine (Mediatech Inc., Manassas, VA, USA) supplemented with 8% fetal bovine serum (FBS) and antibiotics (neomycin, 90 U/ ml; gentamicin, 50 μg/ ml; penicillin, 455 I.U/ ml; streptomycin 455μg/ ml; and fungizone, 1.5μg/ ml). The inoculated cells were incubated at 37°C under 5% CO_2_. The cells were examined daily under an inverted microscope for the appearance of cytopathic effects (CPE), which usually appeared 2–3 days after infection. Infected cells were frozen and thawed three times followed by centrifugation at 2,000 × g for 30 min at 4°C. The virus-containing supernatant was decanted and distributed in 1×3 ¼ ultracentrifuge tubes (Beckman Instruments Inc., USA). One ml of sterile 30% sucrose in distilled water was under-laid in each tube. Balanced capped tubes were then ultra-centrifuged at 111,857 × g/ 2.5 hrs/4°C (OptimaTM L-90K ultracentrifuge, Beckman Instruments) using SW 32 Ti swinging bucket rotor. The supernatant was removed carefully and discarded. The virus pellet was re-suspended in 1 ml of sterile distilled water (DW). The purified virus was aliquoted in small amounts and stored at -80°C until used.

### Effect of CAP exposure on FCV infectivity to CRFK cells

To study the effect of CAP exposure on FCV infectivity on the host cell (CRFK), 50% tissue culture infectious doses (TCID_50_) of CAP-treated and untreated were determined and then compared. Briefly, aliquots (100μl) of FCV suspended in distilled water (DW) were distributed in wells of 96-well microtiter plates followed by exposure of triplicate wells to CAP jet under the operational conditions mentioned above (see plasma generation setup details) for various exposure times (15, 30, 60, and120s). Then, the titer of the infectious virus in all CAP treated samples versus the control (CAP-untreated) were measured by the end point cell culture infection technique to determine the 50% tissue culture infectious dose (TCID_50_) as described previously [16, 28, 29]. Briefly, serial 10-fold dilutions of the samples were prepared in MEM containing 4% FBS. Each dilution was inoculated in CRFK cell monolayers in 96-well microtiter plates using three wells per dilution and 100μl of the dilution per well. The plates were incubated at 37°C under 5% CO_2_ and examined daily for the development of CPE for up to five days. The endpoint was taken as the highest dilution of the virus which produced CPE in 50% of the inoculated cells. Viral titers, calculated by the Kärber method [30], were expressed as log_10_ TCID_50_/0.1 ml. Using a microscopic camera, pictures of the cell monolayer and CPE were taken every 4 h post infection for up to 24 h and every day after the first day post infection.

### Observing the destruction of FCV capsids after CAP exposure by transmission electron microscopy (TEM)

The TEM examination was performed by Prof. Anibal Armien, Veterinary Diagnostic Laboratory, University of Minnesota. Aliquots of FCV (100μl) suspended in DW were distributed in wells of 96-well microtiter plates. Triplicate wells were exposed to CAP jet under the operational conditions mentioned above for 15 s exposure time. A pool of 10 samples (1 ml total volume) was prepared and examined by negative contrast transmission electron microscopy [31]. Briefly, 1 ml of CAP treated and 1 ml of untreated (control) samples were transferred separately by pipetting from the microtiter plate into 1.8 ml centrifuge tubes (Eppendorf, Hamburg, Germany) followed by centrifugation at 3000 ×g (Eppendorf, Hamburg, Germany). The supernatants were transferred to fresh centrifuge tubes (Beckman Coulter, Brea, California, USA) followed by centrifugation for 10 min at 30 PSI in an airfuge (Beckman Coulter, Brea, California, USA). The supernatant was discarded and the pellet was re-constituted in 10μl of sterilized Millipore water. The sample (5μl) was placed on parafilm followed by placement of formvar-coated copper grids (Electron Microscopy Sciences, Hatfield, PA, USA) on top of the sample drop for 10 min. Excess liquid was wicked and the grids were stained with 1% phosphotungstic acid (Electron Microscopy Sciences, Hatfield, PA, USA) for a minute. These samples were visualized using a transmission electron microscope (JEOL LTD, Tokyo, Japan). Images were obtained using a Veleta 2K×2K camera with iTEM software (Olympus SIS, Munster, Germany).

### Quantification of damaged and intact FCV capsids using qRT-PCR coupled with ethidium monoazide (EMA-RT-qPCR)

We employed the EMA-RT-qPCR method to quantify FCV with damaged and intact capsids described by Sangsanont et al. [32]. Briefly, 100 μl aliquots of DW-suspended FCV were exposed to plasma for 15 s and 120 s, then mixed with 1μl of ethidium monoazide (EMA dye) (phenantiridium, 3-amino-8-azido-5-ethyl-6-phenyl bromide; Invitrogen, USA) to achieve a final concentration of 5μg EMA/100 μl, and then incubated at 4°C for 30 min in dark. The samples were then exposed to light for 5 min in an ice bath using a 650W halogen bulb (OSRAM GmbH, Munich, Germany). Exposure to light was indirect; by holding the tube on its side to increase the exposure area and to expose it to light through the plastic to avoid the effect of UV. Viral RNA was extracted from the whole sample volume (100μl) using QIAamp® Viral RNA Mini kit (Qiagen, Valencia, CA). PCR-inactive RNAs were quantified by RT-qPCR determination. The titer of FCV with undamaged capsids equals the titer of CAP-treated virus quantified by EMA-RT-qPCR. The titer of FCV with damaged capsids was calculated by the difference between the titer of control and CAP-treated virus as measured by the EMA-RT-qPCR. In addition, conventional RT-PCR reactions were carried out to compare the RNA of plasma-exposed and unexposed virus qualitatively on the basis of their agarose gel patterns. We chose to use EMA-RT-qPCR method over RNase-RT-qPCR because we had a concern that the enzymatic protein of RNase might be affected by the reactive species of CAP and its activity might be disrupted.

#### Quantitative real time RT-PCR (rRT-qPCR)

The real-time RT-qPCR primer set [rRT-forward (5’-CAA CCT GCG CTA ACG-3’) and rRT-reverse (5’-TCC CAC ACA GTT CCA AAT T-3’)] and TaqMan-TAMARA probe (6-FAM-CTT AAA TAY TAT GAT TGG GAY CCC CA-TAMRA) were used (Table 1) as described by Chander et al., [33]. They were designed to cover the region from nucleotide 5321 to 5471 of the viral genome with amplicon size of 151bp. One-step RT-PCR Kit (Qiagen, Valencia, CA) was used. The reaction mixture (20μl) contained 2μl of RNA and 5U of RNase inhibitor (Invitrogen). Reverse transcription was performed at 50°C for 30 min. Taq activation was done at 95°C for 15 min followed by 50 amplification cycles using a 95°C 15s denaturation step and an annealing/extension step at 50°C for 60s. Real-time RT-qPCR was performed in an Eppendorf Mastercycler ® ep Realplex2 thermocycler (Eppendorf, Hamburg, Germany). Fluorescence was measured during the annealing/extension step of each cycle. Viral RNA extracted from 100μl of FCV with a known titer was diluted 10-fold serially in RNase-free water and these dilutions were used to construct a standard curve by conducting real-time RT-qPCR from each RNA dilution. The standard curve translated the cycle threshold (Ct) values into viral titer equivalents (log_10_ TCID_50_/100μl). The standard curve was generated from average Ct values of three measurements per dilution. At each real-time RT-qPCR measurement, a standard curve (average of duplicate standard serial dilutions) was generated under the same conditions as the tested samples.

**Table 1:** Forward and reverse primers with size and annealing temperatures. The position of gel-based RT-PCR primers are based on USDA Feline calicivirus sequence (GenBank accession AY560118.1).

Primer set #	Primer name	Primer sequence (5’-3’)	Sequence region	Annealing temperature	Covered region	Amplicon size (bp)
1	FCV-F1	GTAAAAGAAATTTGAGACA	1–19	50°C	1–1170	1170
	FCV-R1	GCTGCAAGTTTTTGGGTAGC	1151–1170			
2	FCV-F2	TGGAAAGGATTGGTGTGTCA	546–565	52°C	546–1499	953
	FCV-R2	CAGCTGTTGTCTTCCCACAA	1480–1499			
3	FCV-F3	CCCACAACCACATCTGACAC	1250–1269	52°C	1250–2332	1082
	FCV-R3	TGCAGATTGAACGCAGTTTC	2313–1332			
4	FCV-F4	TAACTCCATTGGCTGCACTG	2206–2225	52°C	2206–3247	1041
	FCV-R4	AGTACCAGGGCCAGATTCCT	3228–3247			
5	FCV-F5	TACGCATCAAGGTTTAGGCC	2870–2889	52°C	2870–3850	980
	FCV-R5	CTCAGTATGTGCTGGCGAAA	3831–3850			
6	FCV-F6	ATGACCCAACAAAGCCTGAC	3738–3757	54°C	3738–4662	924
	FCV-R6	GATGTTAGGGGCATTCCAGA	4643–4662			
7	FCV-F7	AGCGAACCACCAGTATGGAC	4363–4382	52°C	4363–5362	999
	FCV-R7	GGGGGTCCCAATCGTAGTAT	5343–5362			
8	FCV-F8	TTGCTTGTACGCCAGTCAAC	5095–5114	50°C	5095–6017	919
	FCV-R8	ACAATGGCAGCCAACTTACC	5998–6017			
9	FCV-F9	TCGACTTGAGGCTGATGATG	5776–5695	52°C	5776–6831	1055
	FCV-R9	GGTGGGGTCTATCACATTGG	6812–6831			
10	FCV-F10	CCAACCGTCACTTCGACTTT	6422–6441	54°C	6422–7464	1042
	FCV-R10	CTTGGTTGTTCAGGGCTTGT	7445–7464			
11	FCV-F11	GGCAACCATCCCATCTTTTA	6994–7013	56°C	6994–7681	669
	FCV-R11	CCCTGGGGTTAGGCGCTAG	7663–7681			
rRT-Forward primer	FCV-1_rrtF (+)	CAACCTGCGCTAACG	5321–5335	52°C	5321–5471	151
rRT-Reverse primer	FCV-1_rrtR (−)	TCCCACACAGTTCCAAATT	5453–5471			
Probe	FCVGG1p	6-AMCTTAAATAYTATGATTGGGAYCCCCA-TAMRA	5339–5363			

#### Conventional RT-PCR

The primer set# 5 (Table 1) [FCV-F5 (5’-TAC GCA TCA AGG TTT AGG CC-3’) and FCV-R5 (5’-CTC AGT ATG TGC TGG CGA AA-3’)] was used for conventional RT-PCR. The primers were designed to cover the region from nucleotide 2870 to 3850 of the viral genome with amplicon size of 980bp. Amplification reactions (25 μl reaction mixture) were performed in an Eppendorf thermocycler (Mastercycler® Eppendorf, Hamburg, Germany) under the following conditions: reverse transcription step was carried out at 50°C/30 min 95°C /15 min followed by 40 cycles of 1 min at 94°C (denaturation); 1 min at the annealing temperature corresponding to the used primer (Table 1), and 1 min at 72°C (extension) and one final extension step of 10 min at 72°C. The PCR products were detected by 1.2% agarose gel electrophoresis in Tris- acetate-EDTA buffer with ethidium bromide.

### Mass spectrometry to determine the oxidative impact of CAP species on capsid proteins

#### A) Proteolytic digestion of capsid proteins

Triplicate samples of plasma-exposed and unexposed FCV were separated on 4–15% gradient (Criterion^TM^ TGX Precast Gels, Biorad, CA) of sodium dodecyl sulfate poly acrylamide gel electrophoresis (SDS-PAGE). The peptide bands on the gel were stained with Imperial Protein Stain (Thermo Scientific, Rockford, IL). Two gel regions from each lane (an upper region ~ 50–74 kDa and a lower region ~27–43 kDa) were excised followed by in-gel trypsin digestion using a trypsin solution [5 ng/μl sequencing grade trypsin (Promega, Madison, WI) in 50 mM ammonium bicarbonate pH 7.8, 5 mM calcium chloride] [34]. The hydrolysates were desalted by using the Stage Tip protocol [35].

#### B) Mass spectrometry

Triplicate samples of peptides (~1 μg) were injected and analyzed by capillary liquid chromatography (LC)-mass spectrometry (MS) on an OrbitrapVelos MS system (Thermo Fisher Scientific, Waltham, MA) using MS1 (survey) scan mode. The parameters of the MS analysis and LC gradient profile are included in S1 File. Label-free relative peak quantification was performed by comparing peak intensities in the following fashion: control upper region compared to upper region of 15s plasma exposed; control upper region compared to upper region of 120s plasma exposed; control lower region compared to lower region of 15s plasma exposed; and control lower region compared to lower region of 120s plasma exposed. The four “treatment” samples (15s upper region, 15s lower region, 120s upper region, and 120s lower region) were analyzed by LC-MS/MS on Orbitrap Velos with HCD (higher energy collision induced dissociation) activation, with precursor ions selected from inclusion lists that were populated after label-free quantification (LFQ). Each sample had a unique inclusion list generated from the LFQ analysis and the samples were analyzed by LC-MS on the same LC column prior to injection of any unrelated samples. Approximately 1 μg of peptide per sample was injected with LC parameters identical to the MS1-only analyses. The MS parameters [36] were used with some modifications as shown in the S1 File.

#### C) Quantification analysis and tandem MS data analysis

Label-free relative quantification of analytes/peptides between control and exposed samples was done with in-RIPPER software [37]. Briefly, median intensity values of control vs. exposed samples were analyzed by the Student's t-test and p-values were calculated. For each comparison, analytes with statistically different intensity values were populated into an 'inclusion' list for the four samples. The inclusion list contained mass/charge values (i.e., candidate peptide peaks) for candidate peptides with differential expression. The inclusion list was incorporated into the MS acquisition method in order to target the peptide candidates for tandem MS analysis. Each analyte was allowed a retention time window of ± 2 minutes from the original quantification analysis to trigger MS/MS fragmentation. The samples were reinjected for the purpose of acquiring tandem MS on peptides of interest that had differential expression between control and exposed samples. The supplemental dataset (Appendix 2) shows ‘area under the curve’ (AUC) (peptide intensities) for each peptide that was identified by PEAKS with the directed inclusion list method, in addition to the p-values from the student's t-test. For peptide spectral matching and protein inference, the MS/MS data triggered from the directed inclusion lists were analyzed in PEAKS® Studio 7.0 build 20140912 software (Bioinformatics Solutions, Waterloo, ON Canada) [38]. All tandem MS spectra that matched the candidate oxidized peptides were manually inspected for quality and only high quality spectra were included; the parameters of analysis are included in Appendix 1. Identifications from PEAKS® Studio 7.0 were matched to RIPPER quantification analysis using an R script developed at the University of Minnesota [37]. Matching criteria included m/z value (±Δ 0.005) and retention time (±Δ 2 min). When duplicate analytes were generated, analytes with the smallest retention time difference were chosen.

### Effect of CAP exposure on viral RNA

Viral genome integration test (described below) was conducted to study the effect of CAP species on viral RNA. In this experiment we tested genome integration of three viral RNA samples, **i)** an RNA sample (referred to as “packed RNA”) in which, the RNA was isolated from FCV **after** the virus was exposed to CAP for 15 s and 2 min, **ii)** an RNA sample (referred to as “unpacked-RNA sample”) isolated in pure form from FCV and then exposed to CAP directly for 15 s and 2 min while it was unprotected by capsid protein, and **iii)** a control (unexposed to plasma) RNA sample.

#### A) Viral genome integration test

We studied the integration of FCV genome by comparing RT-PCR products of the CAP-treated unpacked viral RNA and CAP-treated packed viral RNA (RNA isolated from FCV after CAP treatment). RNA samples compared to CAP untreated RNA (control). Eleven primer pairs (Table 1) covering the entire viral genome were used in RT-PCR to amplify the viral genome in 11 different amplicons. We compared the agarose gel patterns of the two treated samples with control in terms of the presence or absence of bands representing each genome region. All obtained bands were then purified, sequenced and aligned with the control RNA sequence to evaluate the integration and alterations in the sequence.

#### B) Measuring the effect of CAP exposure on viral RNA at longer exposure time

**A**liquots (100 μl each) of FCV suspended in sterilized Millipore water containing RNase inhibitor (10 U/ml), and aliquots (100 μl each) of pure RNA sample (unpacked RNA) isolated from FCV in sterilized RNase-free water containing10 U/ml RNase inhibitor were placed in 96-well plates (100 μl/well) and then exposed to plasma for 15, 30, 60, 120, 180, and 240s. Then packed RNA was extracted from CAP-exposed FCV samples. In both packed and unpacked RNA samples treated with CAP for different exposure times, One-Step RT-PCR reactions were carried out using QIAGEN® One Step RT-PCR Kit (Qiagen, Valencia, CA) and FCV primer set number 5 only (Table 1) at 52°C annealing temperature. The RT-PCR program was 50/30 min, 95/15min, 94/1 min, 52/1 min, 72/1 min for 40x cycles. Equal volumes of each PCR product (8 μl) were used for agarose gel electrophoresis. The intensity and presence or absence of the amplicon (980bp) were compared between the CAP-exposed packed and unpacked viral RNA samples.

### Whole genome sequencing of plasma-exposed and unexposed FCV

#### A) Production of cDNA

Aliquots (100 μl) of DW-suspended FCV were exposed to CAP for 2 min followed by incubation at room temperature for 2 hrs. Viral RNA was extracted and One-Step RT-PCR reactions were carried out to amplify directly the complete genome of plasma-exposed and unexposed FCV by primer walking using a set of specific primers (Table 1) which were designed based on the complete genome sequence of *Feline calicivirus* isolate USDA (accession no: AY560118.1) using Primer3 software. Amplification reactions (25 μl reaction mixture) were performed in an Eppendorf thermocycler (Mastercycler® Eppendorf, Hamburg, Germany) under the following conditions: reverse transcription at 50°C/30 min 95°C /15min, 40 cycles of 1 min at 94°C (denaturation); 1 min at the annealing temperature corresponding to the used primer (Table 1), 1 min at 72°C (extension) and one final extension step of 10 min at 72°C. The PCR products were detected by 1.2% agarose gel electrophoresis in Tris- acetate-EDTA buffer with ethidium bromide.

#### B) Purification and sequencing of PCR products

The PCR products were purified using QIAquick PCR purification kit (Qiagen, Valencia, CA) followed by submission to the University of Minnesota Genomics Center (UMGC) for sequencing. The same forward and reverse primers were used as in PCR reaction with the dideoxy chain termination method and primer walking strategy. The obtained sequences were curated and aligned using “Sequencher 5.1” software (https://genecodes.com) followed by BLAST analysis in GenBank database. The compatible nucleotide (nt) sequences were aligned by using the Clustal W option in MEGA 6.0 (Molecular Evolutionary Genetic Analysis) to obtain a consensus sequence. The variations in plasma-treated and untreated viral genome were analyzed by P distance option in Mega 6.0 based on [39].

### Statistical analysis

For virus titration using TCID_50_ method on cell culture and for titration using the EMA-RT-qPCR method, the results are the average of triplicate experiments for each treatment. Error bars on some figures represent the standard deviation. The significance of differences between treatments was statistically tested by the one-way analysis of variance (one-way ANOVA) using F-test. For multiple comparison between means, we used the Fisher’s least significant difference (Fisher’s LSD) test. The statistical analysis was performed using STATISTICA software package, version 10 (Statsoft Inc., Tulsa, OK, USA). The statistical analysis of the mass spectrometry study was explained in the material and methods section and in S1 File.

## Results and discussion

Feline calicivirus consists of a single stranded RNA packaged in an icosahedral capsid. The capsid is constructed of 180 units of a single major capsid protein (referred to as VP1) arranged in 90 VP1 dimers as shown by x-ray crystallography shown in S1 Fig [40–41]. The VP1 consists of three domains; (i) an N-terminal arm (NTA) (peptide residues 129–168 of the capsid’s amino acid sequence); (ii) a shell (S) domain (peptide residues 169–329); (iii) and a protrusion (P) domain (peptide residues 330–662). The latter is divided into P1 and P2 subdomains; the P1 subdomain exists in two polypeptide segments: pps-1 (residues 330–381) and pps-2 (residues 551–662) while the P2 subdomain (the outmost protein of capsid) is inserted between pps-1 and pps-2 (residues 381–550) [41, 42]. Capsid of FCV and its major capsid protein (VP1) have a structure similar to those of the capsid of other members of *Caliciviridae* including human noroviruses (HuNoVs) with the same domain arrangement as seen by x-ray crystallography [40–42]. However, the receptor binding sites on HuNoVs capsid are different from on FCV, which limits the possibility of extending these conclusions to norovirus.

### Effect of CAP treatment on the infectivity of FCV

**Fig 2:**
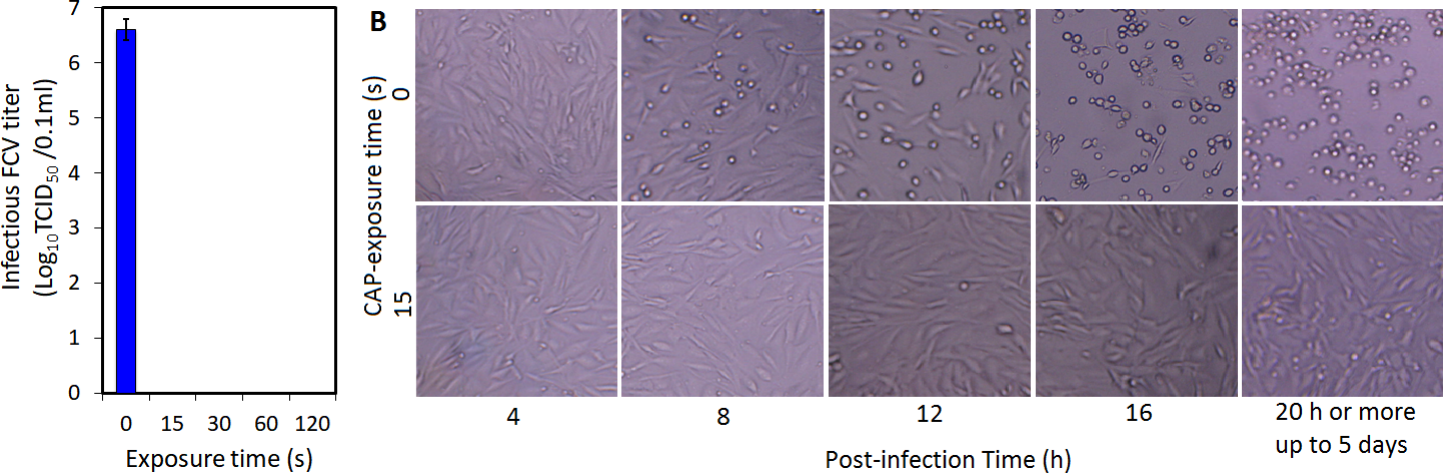
CAP exposure effect on FCV infectivity. **A)** Infectious FCV titer before and after exposure to CAP for 15, 30, 60, and 120s. **B)** Inverted microscope image (magnification power 40 X) of CRFK cells infected with CAP-unexposed and CAP-exposed (15s exposure) FCV at different post-infection times up to 5 days. Serial of 10-fold dilutions from triplicate samples of CAP-exposed and unexposed FCV were prepared separately. Each dilution was inoculated in CRFK cell monolayers (three wells per dilution) and then incubated at 37°C under 5% CO_2_ for 5 days. The number of positive wells showing CPE was recorded for each sample and the infectious virus titer was calculated by the Karber method [30]. Columns are the average value of triplicate samples and the error bars represent the standard deviations.

As shown in Fig 2A, the titer of infectious FCV in the control sample (CAP-unexposed) was ~6.5 log_10_ TCID_50_/100μl. After exposure to CAP for 15s or more, all infectious FCV was completely inactivated. The light microscopy images (Fig 2B) showed that no CPE formed in CRFK cells infected by FCV exposed to CAP for 15 or more even after 5 days post infection. This result indicates that FCV exposed to CAP completely lost its infectivity at a very short exposure time (15s). To understand the impact of CAP exposure on FCV we tested the changes in the viral capsid by TEM examination since the breakdown and oxidation of viral capsids by species of CAP have been suggested in other viruses such as bacteriophage MS2 [19], bacteriophage lambda and adenovirus [18] and adenovirus [20].

### Transmission electron microscopy (TEM)

**Fig 3:**
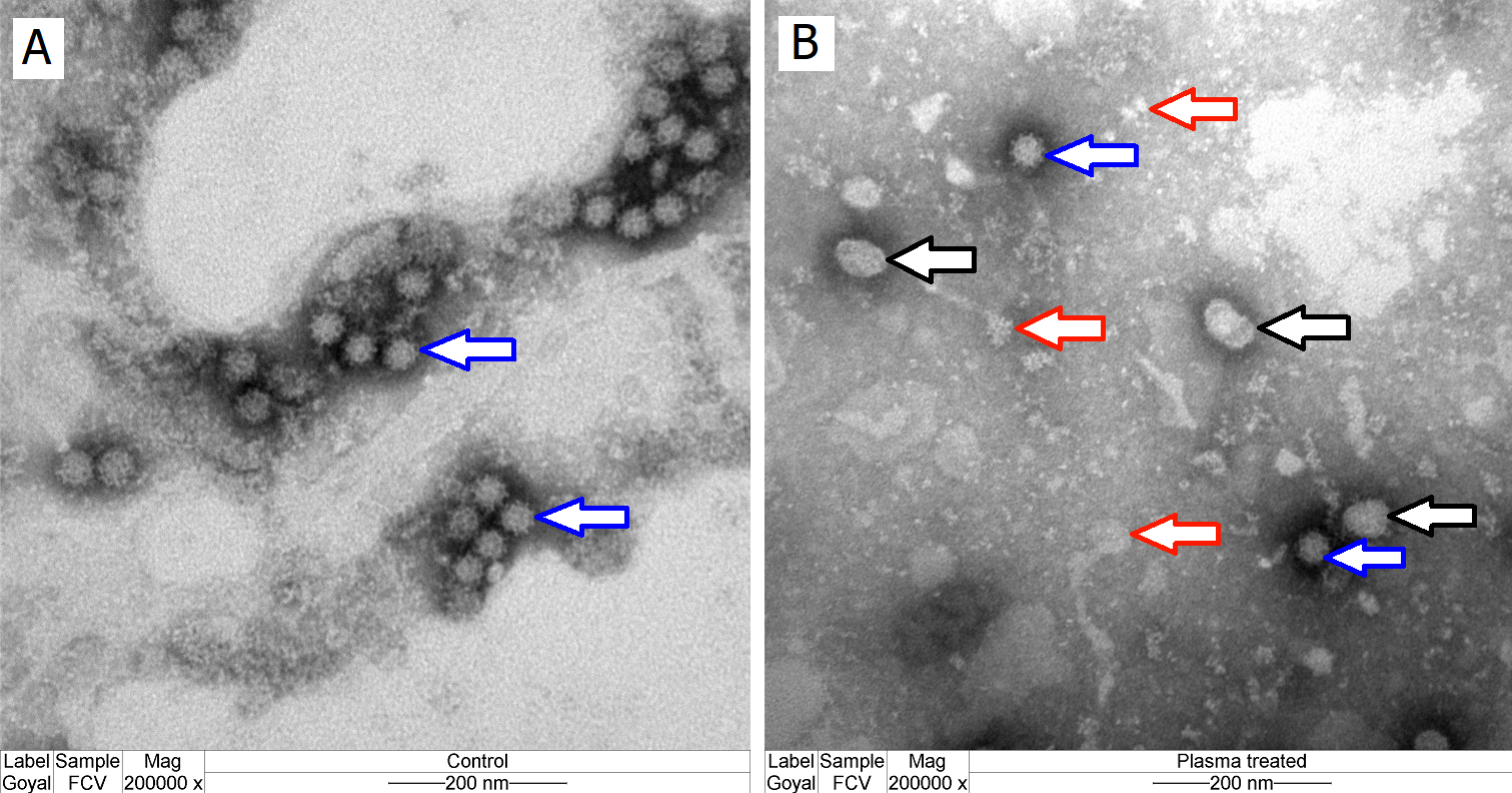
**Transmission electron microscopic images of FCV (magnification 200,000 X): A)** CAP-unexposed virus; arrow indicates virus particles). **B)** CAP-exposed for 15s (black arrows refer to distorted viral particles; red arrows to debris of plasma-affected viral particles; and blue arrow to intact virions).

The control FCV particles appeared intact under TEM (Fig 3A, blue arrow) while 15s plasma-exposed FCV showed destruction of a majority of viral capsids as manifested by the presence of debris and disintegrated pieces of the virus capsid (Fig 3B, red arrows) in addition to distorted capsids (Fig 3B, black arrows). Only a few intact virus particles (Fig 3B, blue arrows) were observed in plasma-exposed FCV indicating that the viral capsid protein is the main target of the reactive species in liquid treatment. This result is consistent with the electron microscope image that showed strong capsid damage in bacteriophage MS2 (nonenveloped RNA virus) by CAP exposure [19]. However, destruction of viral capsid alone does not explain the complete inactivation of the virus, because all virions are not destroyed, indicating that some other mechanisms is also operative. The fraction of FCV with undamaged capsids was quantified to determine if they play a significant role in virus inactivation. Since TEM cannot be used for accurate quantification of intact viral fraction, we used the EMA-RT-qPCR method.

### Quantification of FCV with damaged and intact capsids using EMA-RT-qPCR

**Fig 4:**
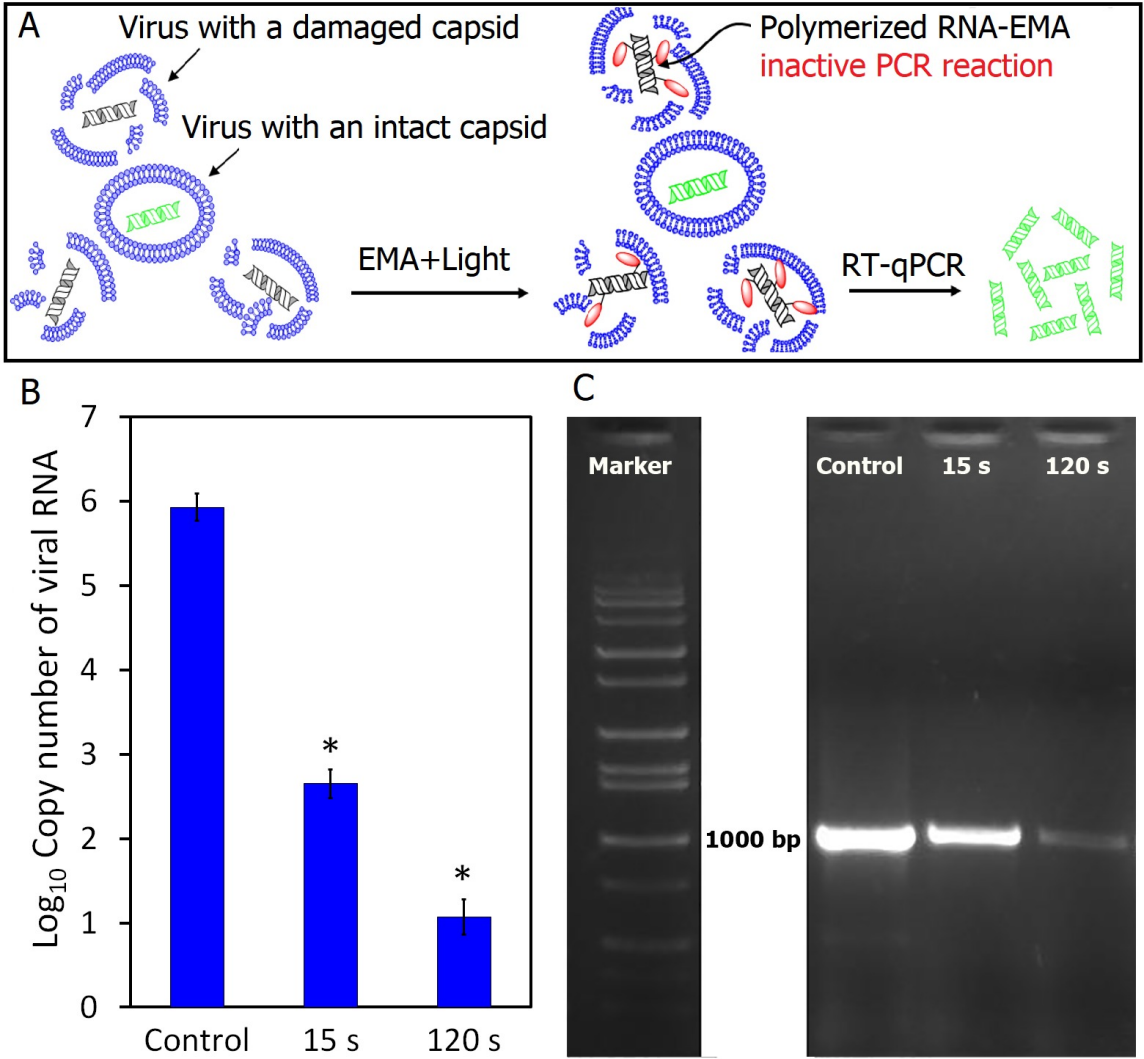
Quantification of capsid-destruction as a function of CAP-exposure time. **A)** Diagram explaining the principle of RT-qPCR coupled with ethidium monoazide (EMA-RT-qPCR). **B)** The titer of capsid-intact FCV after 15 and 120 s exposures to CAP as compared to control (CAP-unexposed). The data are average of triplicate measurements and error bars represent standard deviation. Labels on top of columns refer to the significance of RNA concentration as compared to control of each sample [*: Statistically significant (P≤0.05)]. **C)** The agarose gel patterns of EMA-coupled conventional RT-PCR products generated from viral RNA obtained from 15s and 120s CAP-exposed and unexposed FCV samples. Aliquots (100 μl) of 15s and 120s CAP-exposed FCV were mixed with 5μg EMA/100 μl followed by incubation at 4°C for 30 min in dark. Samples were then exposed to light (using a 650W halogen bulb) for 5 min in ice bath followed by viral RNA extraction. Quantification of PCR-reactive and PCR-nonreactive RNAs in control and CAP-exposed samples was carried out by RT-qPCR. Conventional RT-PCR reactions were carried out using RNA extracted from control and CAP-exposed samples to compare them qualitatively based on agarose gel patterns of a region of viral RNA nucleotide.

The EMA-RT-qPCR method described by Sangsanont et al. [32] was used to quantify the number of FCV particles with undamaged capsids. In this method, damaged capsids allow penetration of EMA dye, which react with the viral RNA followed by polymerization upon exposure to light, thus preventing its PCR reactivity **(**Fig 4A). The results showed that the number of PCR-reactive RNA copies in CAP-exposed FCV samples significantly decreased (P<0.05) (~3.3 and ~4.9 Log_10_ genome copy number reduction/100 μl for 15s and 120 s CAP-exposed samples respectively) as seen in Fig 4B. This indirectly means that ~ 2.7 and 1.0 log_10_TCID_50_/100 μl FCV retained intact capsids after CAP exposure for 15s and 120s, respectively. Also compared to the control, the 15s plasma-exposed FCV samples showed bands with reduced intensity on agarose gel when a conventional RT-PCR reaction coupled with EMA treatment was carried out. By increasing plasma exposure time to 120s, a very faint band was obtained (Fig 4C). This also gives a qualitative confirmation of the EMA-RT-qPCR results. Since FCV grows very well in CRFK cells even in low titers, the remaining viral particles with undamaged capsids should have been able to show CPE if they are still infectious to the host cell. However, this was not the case (see Fig 2); which suggests that, in addition to destruction and disintegration of capsids, some other mechanism renders the inactivation of by CAP exposure, perhaps due to oxidation of some functional peptides on capsid protein without distorting the capsid and/or the effect on viral genome. A similar phenomenon was observed when HuNoV was exposed to copper alloy surfaces and high hydrostatic pressure treatment (HHP). Exposing HuNoV VLPs to copper alloy surface for 120 min the majority of VLPs capsids were damaged but ~25% were found intact. However, both damaged and intact capsids of HuNoV VLPs completely lost their binding capability with the host receptors [43]. Similarly, the HuNoV VLPs completely lost their binding capacity to host binding sites after exposure to HHP at 900-MPa for 1 min, but about 10% of the virus capsid protein remained intact [44]. Also, Moore at al [45] observed that VLPs capsids retained their structure after loss of receptor binding (infectivity) when the time was varied at a given temperature of heat treatment. The authors explained that receptor binding is dependent upon higher order protein structure (structure-specific binding) that can be lost even though the capsid is intact [45].

### Oxidative impact of CAP species on capsid proteins

**Fig 5:**
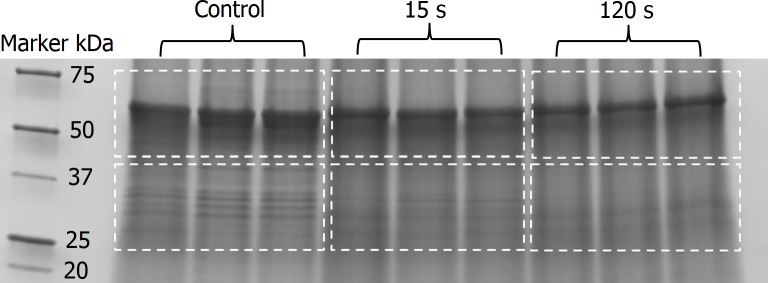
One dimensional SDS-PAGE (4–15% gradient gel) image of CAP-exposed FCV proteins (15 s and 120 s vs. control). The gel was stained with Imperial Protein Stain (Thermo Scientific, Rockford, IL). Two gel regions from each lane were excised; upper region from approximately 50–74 KD and a lower region from approximately 27–43 KD.

To track oxidative modifications in the viral capsid proteins mediated by the reactive species of CAP, LC-MS/MS analysis was performed on the proteins of two SDS-PAGE-separation regions (approximately 50–74 kDa and 27–43 kDa in the upper and lower regions, respectively), which represented the capsid proteins (Fig 5). These two regions were selected for this analysis since the capsid protein of FCV is synthesized as a 76 kDa precursor, which is post-translationally processed into a mature 62 kDa capsid protein by removal of the N-terminal 124 amino acids [46]. Another 40 kDa protein related to FCV capsid protein has also been detected [46]. Fifteen trypsin peptides from capsid protein (the sequence accession gi|692348862 used as reference) were identified by Orbitrap- LC-MS/MS analysis of gel bands, which corresponds to 40.4% coverage in the alignment of the capsid protein (Table 2). Table 3 shows the peptide mass accuracy, peptide identification scores, and the domains in which they are contained within the major capsid protein (VP1) of peptide fragments containing detected or proposed oxidized amino acids. Label-free relative quantification of peptides between control and plasma-exposed samples performed with RIPPER software is shown in Spreadsheet S1.

**Table 2:** Peptide fragments of trypsin-digested FCV capsid protein detected by OrbitrapVelos-MS system using the protein gi|692348862 as a reference sequence.

Position in sequence	Resulting peptide sequence	Peptide length [aa]	NCBI accession number of the FCV capsid protein includes the identical peptide sequence	The amino acids of the reference sequence (gi|692348862) that are different from the corresponding ones (underlined) in the identified sequences
191–206	QSLGPLLNPYL**H**HLAK	16	gi| 4218158	E
231–259	LAAIVVPPGV**R**PVQSTSMLQYPHVLFDAR	29	gi|281376891	N
260–272	QV**H**PVIFSIPDLR	13	gi|2645692	E
322–353	FHLLKPPGSMLTHGS**I**PSDLIPK↓**N**SSLWIGNR	32	gi|66276744	V, S
354–372	HWTDI**NG**F**A**IRPFVFQANR	19	gi|76443954	T, D, V
373–387	HFDFNQETAGW**I**TPR	15	gi|115345268	S
388–398	FRPITITISEK**K**	11	gi|5706699	D
403–428	LGIGVAMDS**V**VPGIPDGWPDTTIPEK	26	gi|2062491	I
429–449	LVPAG**N**YAI**T**NGTGNDITTAK	21	gi|2062485	D, A
450–474	DYDSATVIQNNTN**F**K↓GMYFCGSLQR	25	gi|2062495	I
465–474	GMY**T**CGSLQR	10	gi|5706696	I
494–507	DNTITPSNVIDPTK	14	Identical to the reference	—
560–592	GGNHPIFYK↓NSI**R**↓LGYVIR↓SID**A**FNSQILHTSR	33	gi|2645690	K, V
579–592	S**V**DVFNSQILHTSR	14	gi|115178355	I
639–656	LE**Y**PL**S**ASYMGIQLAKIR	16	gi|305105	F, T

↓ missed cleavage. There should be cleavages at these positions based on the theoretical specificity of the used proteolytic enzyme “trypsin”. However, no cleavages were observed on these positions practically.

Underlines refer to the amino acids in the identified peptide residues that are different from the corresponding amino acids in the reference sequence

The percent coverage based on amino acids detected was 40.4

**Table 3:** Unique peptide fragments containing oxidized amino acids in CAP-exposed FCV capsid protein.

Position in sequence	Resulting peptide sequence (see explanations)	Modified amino acid residue	Mass shift	Theoretical peptide mass [Da], monoisotopic	Peptide precursor *m/z*, monoisotopic	ppm	-10logP score	Treatment/Gel separation region	Domain	NCBI accession number
191–206	QSLGPLLNPYL**H**^**202**^HLAK	His-202	+14	1813.9839	605.6633	-8.6	31.22	15s/upper	S	gi|4218158
231–259	LAAIVVPPGVRPVQSTS**M**^**248**^LQYPHVLFDAR	Met-248	+16	3176.7168	795.1820	-5.6	46.17	15s/lower & 2min /upper	S	gi|281376891
231–259	LAAIVV**P**^**237**^PGVR**P**^**242**^VQSTS**M**^**248**^LQYPHVLFDAR	Pro-237&242; Met-248	+28, +28, +16	3232.7065	809.1879	+4.9	20.90	2min/upper	S	gi|281376891
260–272	**Q**^**260**^V**H**^**262**^PVIFSIPDLR	Gln-260, His-262	-17, +14	1516.8038	759.4071	-2.8	28.78	15s/upper & 2min/upper and lower	S	gi|2645692
322–353	FHLLKPPGS**M**^**331**^LTHGSIPSDLIPKNSSLWIGNR	Met-331	+16	3527.8711	882.9780	+3.3	27.28	2min/upper	S & P_1_ pps-1	gi|66276744
354–372	**H**^**354**^WTDINGFAIRPFVFQANR	His-354	+14	2302.1396	768.3952	+7.0	31.30	2min/upper	P_1_ pps-1	gi|76443954
373–387	HFDFNQETAG**W**^**383**^ITPR	Trp-383	+30	1847.8228	616.9438	-7.1	39.07	15s/upper & 2min/upper &lower	P_1_ pps-1 & P_2_	gi| 115345268
403–428	LGIGVA**M**^**409**^DSVVPGI**P**^**417**^DGWPDTTIPEK	Met-409, Pro-417	+16, +28	2707.3413	903.4639	+10.2	22.74	15s/upper	P_2_	gi|2062491

**-10logP score:** Peptide scores from PEAKS scoring algorithm; > 20 represents 0.5% false discovery rate

**ppm:** Precursor mass error

**S:** Shell domain of the major capsid protein (VP1) of FCV

**P1:** subdomain 1of protrusion (P) domain of VP1

**P2:** subdomain 1of protrusion (P) domain of VP1

**pps-1:** The poly peptide segment one of P1 subdomain

**pps-2:** The poly peptide segment two of P1 subdomain

Underlined peptides were identified without quantification.

**Fig 6:**
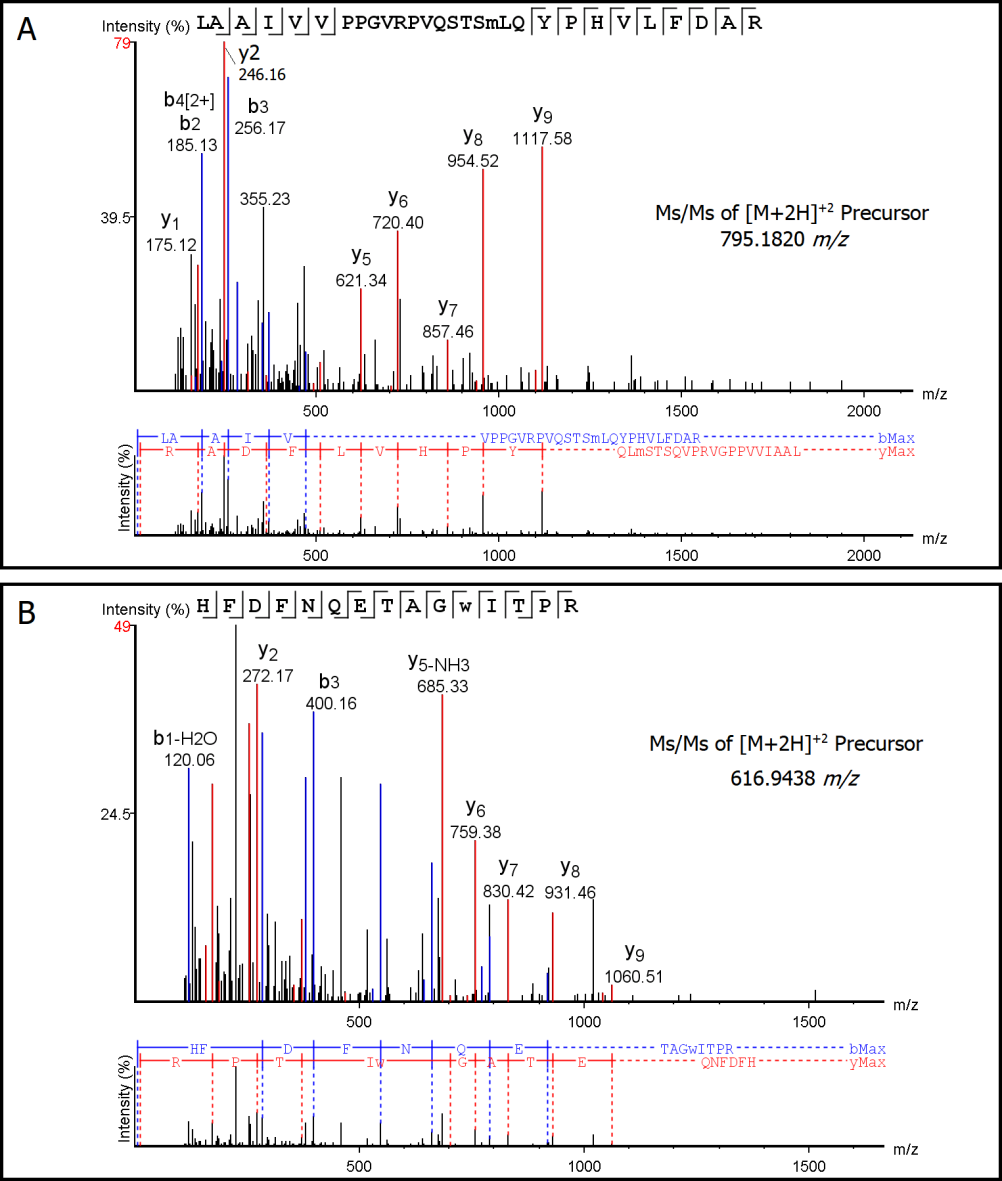
**Representative LC-MS/MS annotated spectrum (top) with alignment (bottom) of**: A) monoisotopic [M + 2H]2+ observed precursor 795.182 m/z matched to peptide LAAIVVPPGVRPVQSTSM(+16)LQYPHVLFDAR PEAKS -10logP score 46.17. **B)** monoisotopic [M + 2H]^2+^ observed precursors 616.9438 *m*/*z* matched to peptide HFDFNQETAGW(+30)ITPR, PEAKS -10logP score 39.07. Theoretical b- and y-type fragment ion types matched to experimental product ion peaks are labeled (spectrum copied and pasted from PEAKS® Studio 7.0). The identified b and y ions are mapped onto the primary sequence and demonstrate modifications in the identified peptides. The peptide scores for the set of representative peptides have a false discovery rate of 0.5%.

Eight unique oxidized peptides were identified in LC-MS/MS spectra of the plasma-exposed sample as compared to those of the control virus (Table 3). Representative tandem mass spectra of unique modified peptides detected in plasma-exposed FCV samples compared to control samples are shown in the Fig 6. The molecular mass of His202, His262, and His354 residues shifted to His+14Da, which is identified as an oxidized His derivative referred to as 2-imidazolone (or 2-oxo-His). The 2-imidazolone (2-oxo-His) modification in His-262 was detected in the monoisotopic [M + 2H]^2+^ peptide precursor peak at *m/z* = 759.4071 matching to peptide [**Q**^**260**^V**H**^**262**^PVIFSIPDLR] in FCV capsid protein sequence. The modifications at His202 and His354 were detected by MS/MS without quantification. The monoisotopic [M + 2H]^2+^ precursor peak 605.6633 *m/z* matched to peptides [QSLGPLLNPYL**H**^**202**^HLAK] and the monoisotopic precursor 768.3952 *m/z* matched to [**H**^**354**^WTDINGFAIRPFVFQANR] as seen in S2 Fig. This oxidative modification of His residues was found to be mediated specifically by singlet oxygen (^1^O_2_) species and was suggested to be a unique marker for singlet oxygen-mediated oxidative stress on proteins [47, 48] as discussed in Aboubakr et al. [17].

The observed molecular mass of methionine residues (Met248, Met331, and Met409) shifted to Met+16 Da, which was identified as methionine sulfoxide. The methionine sulfoxide (Met +16 Da) modifications were detected in the monoisotopic [M + 2H]^2+^ peptide precursor peak at 795.1820, 809.1879, 882.9780, and 903.4639 *m/z*, which matched to peptides [LAAIVVPPGVRPVQSTSM^**248**^LQYPHVLFDAR] (Fig 6A), [LAAIVVP(+28)PGVRP(+28)VQSTS**M**^**248**^LQYPHVLFDAR], [FHLLKPPGS**M**^**331**^LTHGSIPSDLIPKNSSLWIGNR], and [LGIGVA**M**^**409**^DSVVPGIP(+28)DGWPDTTIPEK], respectively.

It has been reported that methionine sulfoxide is an oxidative product of Met mediated by ^1^O_2_ or O_3_ [49–50]. Although methionine sulfoxide may occur as an artifact formed during the preparation of proteins for MS detection [51], the observed methionine sulfoxide cannot be attributed only to this artifact because the level in the plasma-treated virus is significantly higher than in the control as determined by RIPPER analysis. The detection of singlet oxygen-mediated 2-oxo-His and singlet oxygen or ozone mediated methionine sulfoxide as the major oxidative modifications in plasma-exposed viral capsids supports the finding of our previous study in which singlet oxygen in combination with O_3_ were the key reactive species responsible for virus inactivation in solution exposed to plasma [17].

The 30 amu increase in Trp383 corresponds to tryptophandione (Trp+30 Da). This oxidative modification was detected in the monoisotopic [M + 2H]^2+^ precursor peak at *m/z* = 616.9438 matching to peptide [HFDFNQETAG**W**^**383**^ITPR] (Fig 6B). The detection of tryptophandione as an oxidative product of Trp383 suggests the presence of hydroxyl radical as one of the plasma produced species. It was found recently that the oxidative pathway of tryptophan ending in the formation of tryptophandione is specifically mediated by hydroxyl radicals [52]. However, in a previous study we found, through assessing the effect of mannitol (a scavenger of OH) that OH radicals do not play a key role in FCV inactivation [17]. Nonetheless partial suppression of the virucidal effect was found when the FCV was in an NTE solution under the same plasma conditions indicating that OH radicals are produced by the plasma. Since plasma-produced OH radicals only penetrate into the liquid for tens of micrometers before recombining, their production most likely needs to proceed to secondary liquid phase chemistry to significantly impact virus inactivation [2]. Such secondary chemistry is indeed available at pH >5 through peroxone chemistry in the presence of H_2_O_2_ and O_3_. This process was less effective at the reduced pH values in the present study suggesting that the oxidative impact of plasma-produced OH radicals in liquid is present but on its own is unable to cause significant virus inactivation as do O_3_ and singlet oxygen.

The observed modification to Pro237 and the proposed modification to Pro242 are due to carbonyl addition to proline, particularly formyl proline, which corresponds to a mass increase of 28 amu. Both of the modified residues (Pro237 and Pro242) were detected in the monoisotopic [M + 2H]^2+^ precursor peak at *m/z =* 809.1879, which matched with peptides [LAAIVV**P**^**237**^PGVR**P**^**242**^VQSTSM(+16)LQYPHVLFDAR]. In addition, pyroglutamic acid (Gln -17 Da) was detected at Gln260 in peptide [**Q**^**260**^V**H**^**262**^PVIFSIPDLR], with monoisotopic [M + 2H]^2+^ peptide precursor peak at *m/z* = 759.4071. Pyroglutamic acid is an uncommon amino acid derivative in which the free amino group of glutamic acid or glutamine cyclizes to form a lactam. N-terminal glutamic acid and glutamine residues have been found to cyclize spontaneously forming pyroglutamate [53] indicating that the observed modification of Gln260, identified as pyroglutamate, may be spontaneous as it is a terminal Gln residue.

### Impact on capsid functionalities in relation to the oxidative impact

**Fig 7:**
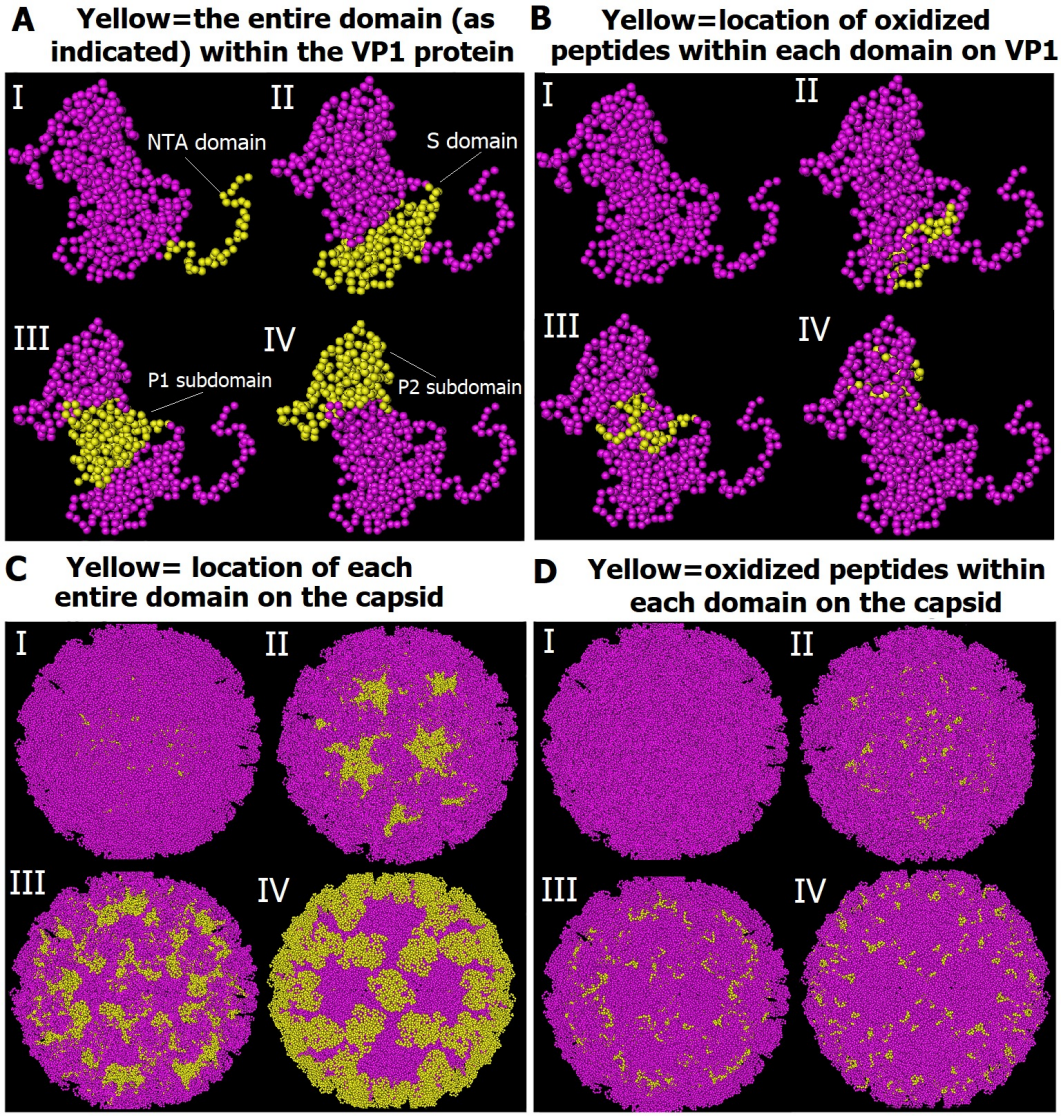
Space-filling model of the structure of the major capsid protein (VP1) and the complete FCV capsid. **A) Location of all domains constructing the VP1protein.** The yellow colored regions represent the whole NTA domain (**I**); the whole S domain (II); the whole P1 subdomain (III); and the whole P2 subdomain (IV). **B)** Location of oxidized peptide residues (yellow colored) among NTA domain (**I**)*; S domain (II); P1 subdomain (III); P2 subdomain (IV). **C)** Location of all VP1 domains within the whole FCV capsid. The yellow colored regions represent the whole NTA domain (I); the whole S domain (II); the whole P1 subdomain (III); and the whole P2 subdomain (IV). **D)** Location of oxidized peptide residues (yellow colored) among NTA domain (I)*; S domain (II); P1 subdomain (III); P2 subdomain (IV). Images were created by Cn3D software version 4.3 using crystal structure of FCV [41] available at: http://www.ncbi.nlm.nih.gov/Structure/mmdb/mmdbsrv.cgi?uid=83065 * No yellow color since there was no oxidized peptide residue detected in NTA domain.

Capsid-related functions such as virus assembly, antigenicity, and receptor interactions are mostly encoded in a single protein in the icosahedral capsid of the virus [54]. Hence, we studied the correlation between structural composition of FCV capsid and the identified oxidative peptide modifications of this structure to determine how these oxidized capsid peptides may lead to FCV inactivation, even without damage to the virus capsid. We analyzed the major capsid protein and images of virions in a space-filling model of FCV’s crystal structures to locate the positions of oxidized peptide residues in the major capsid protein VP1 (Fig 7). As shown in Table 3 and Fig 7, four of the oxidized peptides were located in the S domain; one at the end of the S domain and the beginning of pps-1 of P1 subdomain (exactly at the flexible hinge); one was located in pps-1 of P1 subdomain; one in P2 subdomain; and one at the end of pps-1 of P1 subdomain and the beginning of P2 subdomain. No oxidized peptide was detected in the NTA domain.

The oxidative species of plasma not only affected the outer surface of the capsid (particularly the P2 domain; Fig 7B-IV and 7D-IV), but also the residues that are buried or not easily accessible from the surface of the capsid. These include: the shell proteins (S domain) at an area located between the formed dimers of protrusion proteins (Fig 7D-II) and the hinge region linked between the shell and the protrusions. We believe that this oxidation of the fundamental domains of FCV capsid proteins leads to structural disintegration and distortion of the majority of virions by plasma exposure as observed by TEM (Fig 3).

The feline junctional adhesion molecule A (fJAM-A) is a functional receptor on the host cells (CRFK) that attaches with FCV and plays a role in its entry into the host cell [41, 55]. Also, flexibility in the hinge region is important to permit conformational changes and movement of the capsid’s structural domains in relation to each other during interaction with the fJAM-A receptors [41]. Any change in the flexible hinge’s amino acids, particularly at the amino acid residue Gly329, substantially alters the susceptibility of FCV to attach to fJAM-A [41]. Hence, the detected oxidation in hinge’s peptide residue (Phe322- Arg353), particularly in Mit331 (the second amino acid residue next to Gly329), indicates that oxidation of this peptide might be responsible for hindering attachment of FCV capsid to host cell receptor.

**Fig 8:**
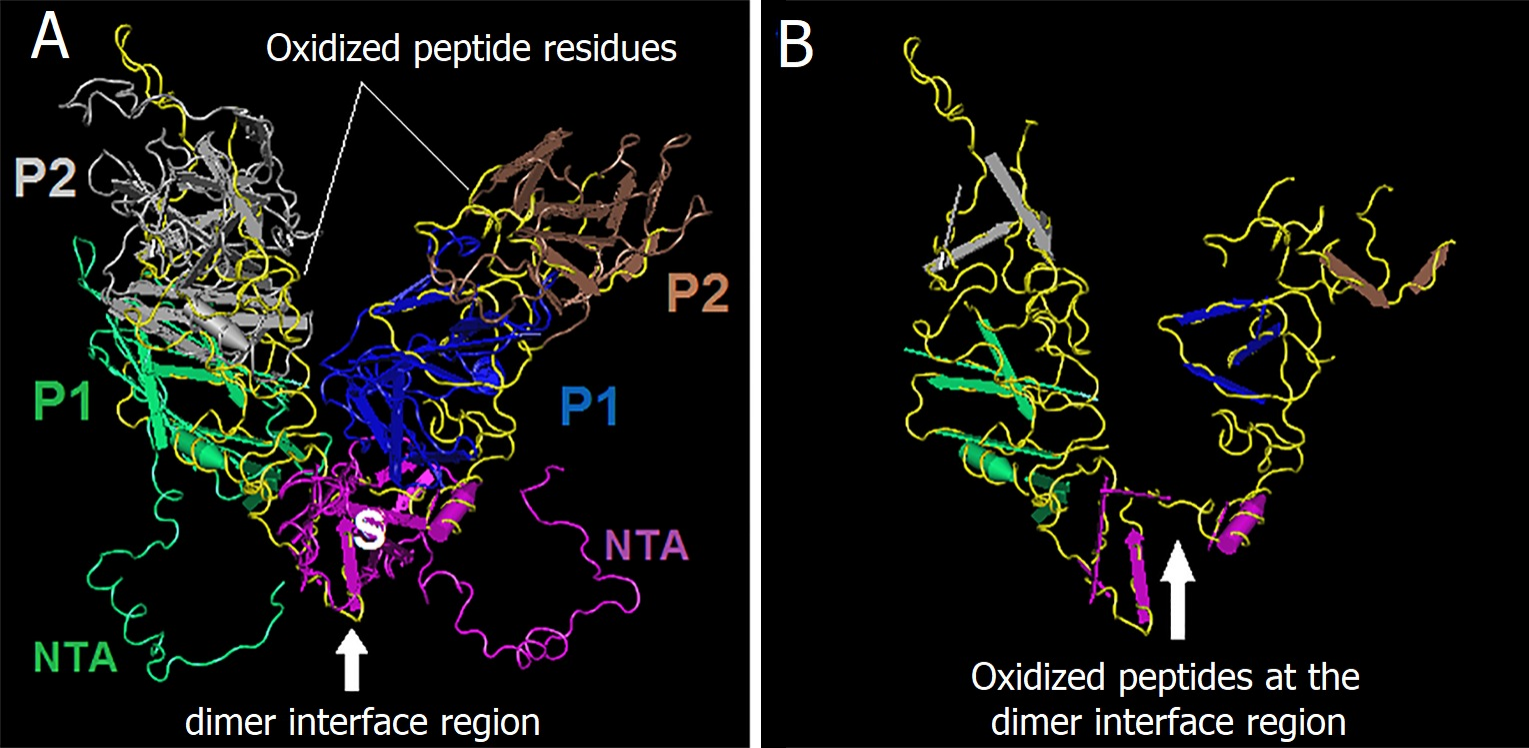
Crystal structure of A/B dimer of the major capsid protein (VP1) using warm model. **A)** The crystal structure of A/B dimer showing the dimer interface region, the domains of VP1 (S, P1, P2, and NTA domains), and the location of oxidized peptide residues (in yellow color) within the crystal structure of A/B dimer. **B)** The crystal structure of the oxidized peptide residues only (yellow colored) virtually separated from the entire A/B dimer. Images were created by Cn3D software version 4.3 using the crystal structure of FCV [41] available at: http://www.ncbi.nlm.nih.gov/Structure/mmdb/mmdbsrv.cgi?uid=83065.

Further, the fJAM-A is known to engage the top of the P2 domain causing a rotation in the P dimer at the dimeric interface between the two capsid monomers [55]. This is believed to cause changes in FCV capsid conformation right after its interaction with fJAM-A, which is important for subsequent interactions of the capsid with the cellular membranes [41]. Any modification in the dimeric interface area of the capsid protein structure, therefore, would lead to the inhibition of virus entry into the host cell. By analyzing the 3D crystal structure of the oxidized peptides within the dimeric interface of VP1protein’s A/B dimers, we found that the oxidized peptides were located in the dimeric interface regions as well as along the whole P1 and P2 subdomains (Figs 8 and 7C). This further indicates that the oxidative effects of plasma species on capsid proteins might hinder the attachment of FCV with its receptors thereby preventing the entry of virus into the host cells. However, this explanation needs to be confirmed. The oxidative stress analysis of CAP on the capsid of FCV agrees with the results of Wigginton et al. [22] on the impact of ozone (one of identified CAP species in our system) on binding of bacteriophage F2 to its host cells. However, our results are contrary to those obtained by Sakudo et al. [14] who found that no alteration in capsid protein of an adenovirus by the species of CAP. This might be due to the differences between structures of adenoviruses and caliciviruses and/or the difference between the CAP species generated by their and our plasma setups. In general, our results provide strong evidence that the inactivation of FCV by CAP is mainly caused by damage and disintegration of the capsid protein and to a lesser extent by the oxidative stress on some functional peptides of the capsid protein VP1.

### Impact of CAP exposure on viral RNA

The interaction of reactive oxygen and nitrogen species (RONS) with nucleic acids is a suggested mechanism of bacterial and viral inactivation by cold plasma exposure through interference with viral gene expression and viral RNA replication [14]. It is thus important to assess if the CAP RNA is a possible target of CAP’s reactive species that might play a role in virus inactivation by CAP. So, we studied the effects of plasma-exposure on unpacked and packed RNA of FCV using genome integration and sequence alignment tests.

### Integration of viral genome after CAP exposure

**Fig 9:**
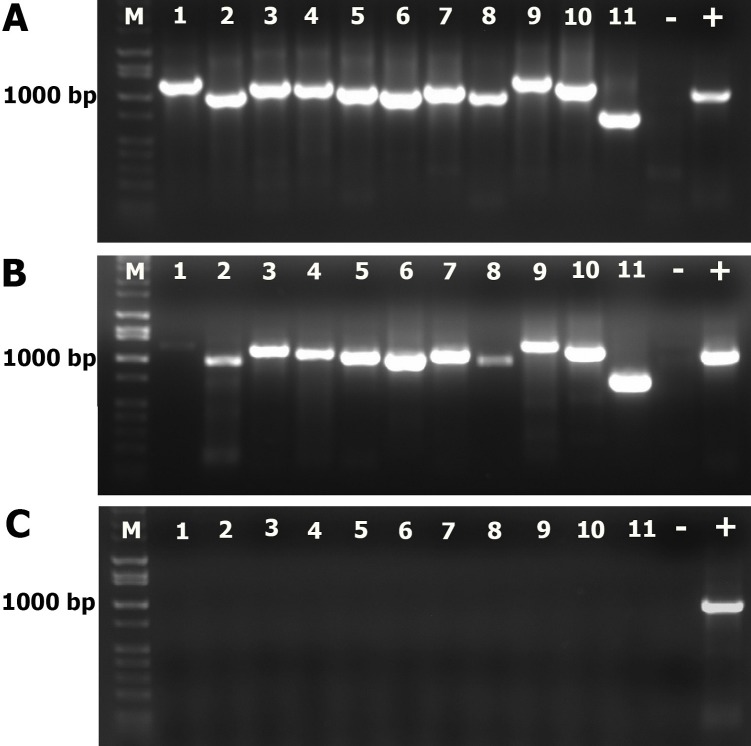
FCV genome integration. **A)** RT-PCR products of RNA isolated from CAP-unexposed FCV. **B)** RT-PCR products of RNA isolated from CAP-exposed FCV for 2 min (capsid-packed RNA). **C)** RT-PCR products of unpacked FCV-RNA exposed for 15 s to CAP. Eleven primer pairs (Table 1) covering the entire viral genome were used in RT-PCR to amplify the viral genome in a total of 11 DNA amplicons.

**Fig 10:**
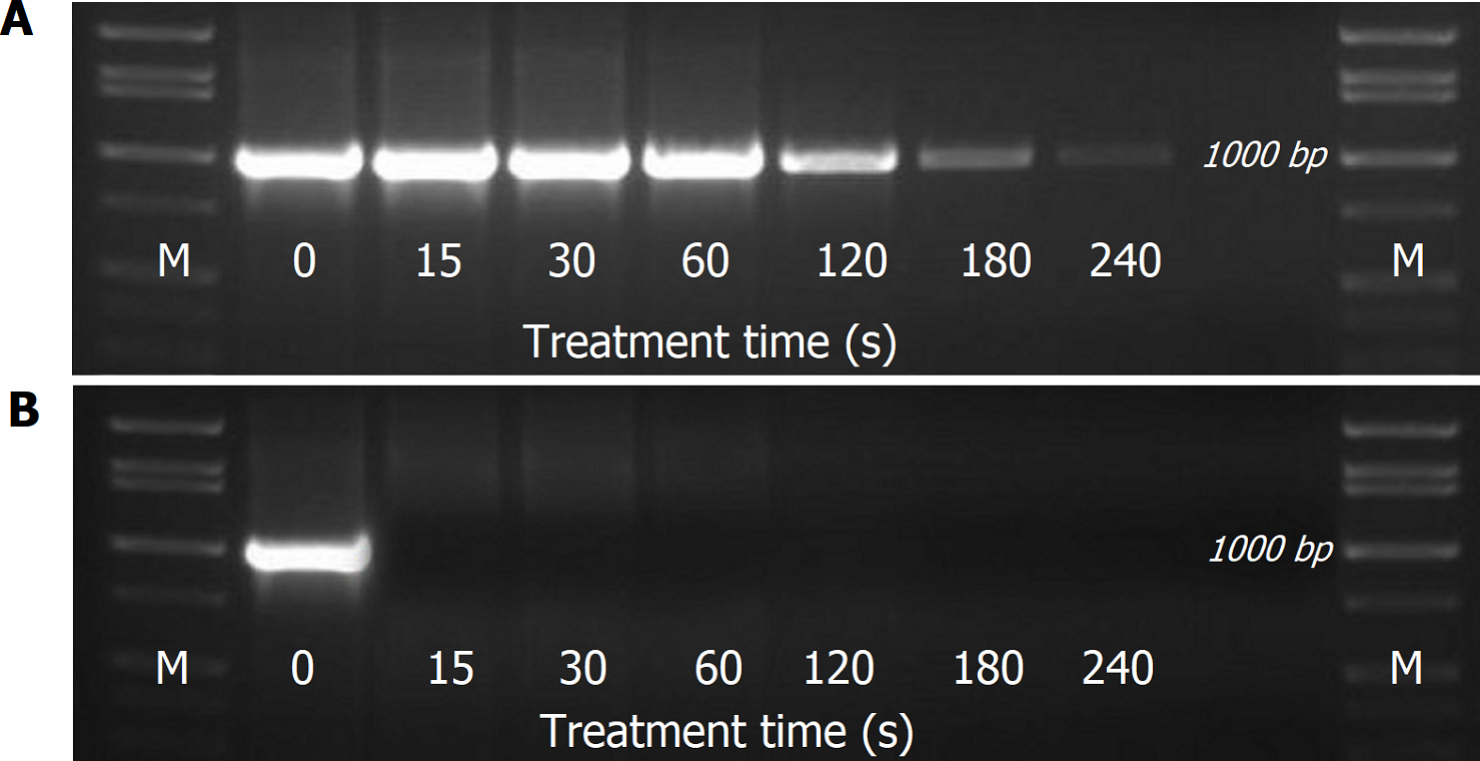
Exposure time dependent effect of CAP on viral RNA. **A)** Agarose pattern of RT-PCR products from **capsid-packed RNA** exposed to CAP (sequence targeted by primer set #5). **B)** Agarose pattern of RT-PCR products from unpacked viral RNA exposed to CAP (sequence targeted by primer set #5). Agarose gel concentration was 1.2% that could separate amplicon sizes from 50–10,000 bp.

The RT-PCR products from CAP-exposed packed-RNA showed agarose patterns identical to the control sample (11 clear bands representing the entire genome regions, Fig 9A and 9B). However, the intensities of the bands of the CAP-exposed samples were lower than the control sample, particularly bands in lanes 1, 2 and 8 were very faint. This indicates that coated RNA was partially affected at the long exposure time (120 s) after being uncoated due to the destruction of capsid. On the other hand, the RT-PCR products from CAP-exposed unpacked-RNA showed agarose pattern free of any DNA band (Fig 9C). This indicates that unpacked viral RNA is very susceptible to CAP species even at short time exposure (15s). However, the presence of RNA inside the viral capsid (packed RNA) partially protects the RNA from the destructive effect of RONS generated by CAP. To see whether exposure times longer than 120 s are able to affect the packed viral RNA or not we performed another test to compare the impact of CAP exposure on packed RNA of FCV at different times (0 to 240s). We used the PCR product of only one representative region on the genome (nucleotide range 2870–3850, 980bp amplicon). The agarose gel pattern of plasma-exposed packed RNA sample showed very clear reductions in the band intensity after 120, 180, and 240s exposure but not for shorter exposure times (Fig 10A), indicating that the capsid protects the packed RNA at short plasma exposure of up to 1 min. At longer exposure time, RNA strands become unpacked because of capsid destruction and are not protected from the oxidative effect of plasma species.

Complete genome sequences of plasma-exposed and plasma-unexposed FCV were deposited in the NCBI Gene Bank under accession numbers KM111170.1 and KM111171.1, respectively. The genome sequencing was done to study the effect of CAP exposure on the viral genome integration and to determine whether CAP exposure leads to modifications in the sequence of viral RNA that remains un-fragmented. Based on the analyses of variation between the genome sequences of the two samples, P distance was zero and the identity was 100%. No deletion, insertion, or variation was observed in any nucleotide. The identical sequences of plasma-exposed and unexposed samples confirmed that plasma exposure did not lead to a significant modification in the viral RNA sequence indicating once again that the oxidative effect of plasma species on the viral RNA takes place only after the destruction of viral capsid and that CAP has no direct impact on viral RNA within the undamaged capsid. These results indicate that the impact of reactive species of CAP on the viral RNA occurs only after the damage and breakdown of the viral capsid. This suggests that the CAP’s impact on viral RNA is secondary to its effect on viral capsid. It is known that the RNA of FCV as well as that of other caliciviruses including HuNoV is infectious by itself. The infectivity of the FCV RNA is due to its linkage with the VPg protein [56], which acts as a primer during RNA synthesis and has a role in translation initiation by acting like a 5' mRNA cap [57]. The impact of CAP on viral RNA presented here is in agreement with Sakudo et al. [14] who indicated oxidation and destruction of adenovirus’ nucleic acid by CAP exposure. Hence, our results are in agreement with the suggestion that the damage in viral nucleic acid is secondary effect to MS2 inactivation by singlet oxygen produced photodynamically [22]

In conclusion, we found that the reactive oxygen and nitrogen species of CAP inactivate FCV mainly by damaging the viral capsid through oxidizing specific amino acids of peptide residues located in the shell (S) and protrusion (P) domains as well as the dimeric interface regions of the major capsid protein (VP1) of FCV. This oxidation of the fundamental structure of FCV capsid leads to a loss of structural integrity and distortion of FCV virions. In addition, the short time exposure of virus to CAP leads to inactivation of the virus by oxidizing specific functional peptide residues located in P2 domain and NTA hinge that are known to play an important role in virus attachment and entry to the host cell. This indicates that short exposure to CAP may inactivate FCV by hindering virus binding to host cell even if it retained an intact capsid. Furthermore, the oxidative impact of plasma species leads to oxidize and damage the viral RNA once it becomes unpacked subsequent to capsid destruction. However, the former effect plays a less important role in virus inactivation since the intact FCV genome as well as other calicivirus are infectious even after uncoated from the capsid.

## Supporting information

S1 File Parameters of LC-MS, parameters of peptide spectral matching and protein inference and optimized parameters of quantification via RIPPER. (PDF)

S1 Fig X-ray structure of FCV capsid. **A)** X-ray structure of FCV viewed along the icosahedral 2-fold axis. Location of a set of A/B and C/C dimers and icosahedral 5-fold and 3-fold axes are shown. **B)** Ribbon representation of the VP1 subunit structure. The NTA (green), S domain (blue), and P1 (red) and P2 (yellow) subdomains are indicated. **Source:** Ossiboff et al., [41].
(TIF)

S2 Fig **LC-MS/MS annotated spectrum (top) with alignment (bottom) of**: **A)** monoisotopic [M + 2H]^2+^ observed precursors 605.6633 *m*/*z* matched to peptide QSLGPLLNPYL**H**^**202**^HLAK, PEAKS -10logP score 31.22. **B)** Monoisotopic [M + 2H]^2+^ observed precursor 768.3952 *m*/*z* matched to peptide **H**^**354**^WTDINGFAIRPFVFQANR PEAKS -10logP score 22.87. Theoretical b- and y-type fragment ion types matched to experimental product ion peaks are labeled (spectrum copied and pasted from PEAKS® Studio 7.0). The identified b and y ions are mapped onto the primary sequence and demonstrate modifications in the identified peptides. The peptide scores for the set of representative peptides have a false discovery rate of 0.5%.
(TIF)

S1 Spreadsheet Label free relative quantification of peptides between control and CAP-exposed samples performed with RIPPER software. (**Spreadsheet A):** Relative quantification of peptides between band 1 of control and 15s CAP-exposed samples. (**Spreadsheet B):** Relative quantification of peptides between band 2 of control and 15s CAP-exposed samples. (**Spreadsheet C):** Relative quantification of peptides between band 1 of control and 120 s CAP-exposed samples. (**Spreadsheet D):** Relative quantification of peptides between band 2 of control and 120 s CAP-exposed samples.
(XLSX)
