# Supplementary material for: Cold argon-oxygen plasma species oxidize and disintegrate capsid protein of feline calicivirus
Source: PLoS One. 2018 Mar 22;13(3):e0194618. doi: 10.1371/journal.pone.0194618 (PMC5864060; doi:10.1371/journal.pone.0194618)
Supplement: S1 File — (PDF) [file pone.0194618.s001.pdf]

Supporting information

S1 File. Parameters of LC-MS, parameters of peptide spectral matching and protein inference and optimized parameters of quantification via RIPPER.

1- Mass Spectrometry

A- MS parameters used in LC-MS in MS1 (survey) scan mode:

Source voltage 1.9 kV, positive polarity, 360 – 1800 *m/z* at 30,000 resolution, 260 °C source temperature, FT AGC 1×10^6, injection time 500 milliseconds.

B-The profile and parameters of LC gradient

330 nl/min; solvent A 98:2, H_2O: ACN, 0.1% FA; solvent B 2:98, ACN:H_2O, 0.1% FA; gradient profile: 0 – 2 min, 2 – 8% B, 2 – 67 min, 8 – 35% B, 67 – 68 min, 35 – 90% B, 68 – 75 min at 90% B.

2- Tandem MS Data Analysis: parameters for peptide spectral matching and protein inference:

A- Data Refine: RAW files were uploaded directly, spectra within 10 ppm precursor mass and 0.2 min retention time were merged, precursor masses were corrected, charge states 2 – 9 were imported, filter quality was >0.65.

B- DE NOVO: parent mass error tolerance 20 ppm, fragment mass error tolerance 0.1 Da, enzyme trypsin, fixed modification carbamidomethyl cysteine (57.0215), variable modification oxidized methionine (15.9949), maximum 3 variable mods per peptide, report 5 peptides.

C- PEAKS Database Search: parent mass error tolerance 50 ppm, fragment mass error tolerance 0.1 Da, enzyme trypsin, precursor monoisotopic, trypsin enzyme, maximum missed cleave sites 2, non-specific cleavage at both ends, fixed mods, variable mods and max number of mods per peptide the same as DE NOVO settings, protein reference database NCBI non-redundant *feline calicivirus* (taxID 11978)from 12/10/14 merged with NCBI RefSeq *Felis* (taxID 9682)from 10/12/12 and contaminants database (http://www.thegpm.org/crap/), false discovery rate estimation Enabled.

D- PEAKS PTM: max number of PTM's per peptide 3, PTM's selected: Deamidation NQ (0.9840), Oxidation DFKNPRYHW (15.9949), Formylation KPRY (27.9949), Acetylation N-term (42.0106), Dihydroxy MFKPRWY (31.9898), Carbamylation K, N-term (43.0058), Cysteic acid C (47.9847), HisImid 13.98 H, Pyro-glutamic acid from Q N-term (-17.0265), Aminotyrosine Y (15.0109), Kynurenin W (3.9949), Oxidation to Nitro WY (44.9851), Hydrated imidazolone H (31.9898), 2-Amino-N-formylureido-succinamic acid H (47.9847), Tryptophandione W (29.9742); **SPIDER** homology match (search for amino acid modifications and *de novo* sequence homology) invoked. We exported peptide summaries with the following PEAKS® parameters: 1% peptide FDR, Protein score (-10logP) 20 and unique peptide 0.

3- Quantification Analysis: quantification via RIPPER Optimized Parameters

RIPPER allows analysts to optimize analyte information extraction from MS1 data. The RIPPER optimized analyte extraction parameters were: Group MZ Distance = .005, Group RT Distance = 120, Minimum Charge to Process = 2, Maximum Charge to Process = 4, Minimum Mass to Process = 100, Minimum Monoisotopic Peak Cluster Size = 2, Minimum Retention Time to Process = 0.0, Maximum Retention Time to Process = 99999, Signal to Noise Ratio = 3.0, Minimum Number of XIC Peaks = 5, XIC mz range = 0.02, XIC Consecutive Peak mz Tolerance = 0.003, XIC Consecutive Peak RT Tolerance = 20.
