# Supplementary figures and images for: Cold argon-oxygen plasma species oxidize and disintegrate capsid protein of feline calicivirus
Source: PLoS One. 2018 Mar 22;13(3):e0194618. doi: 10.1371/journal.pone.0194618 (PMC5864060; doi:10.1371/journal.pone.0194618)

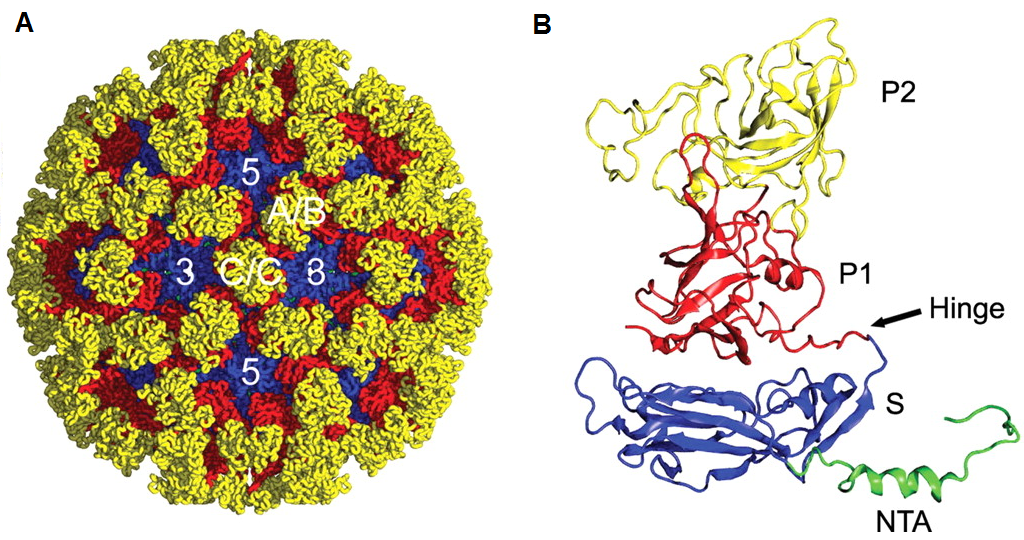

Supplement: S1 Fig — A) X-ray structure of FCV viewed along the icosahedral 2-fold axis. Location of a set of A/B and C/C dimers and icosahedral 5-fold and 3-fold axes are shown. B) Ribbon representation of the VP1 subunit structure. The NTA (green), S domain (blue), and P1 (red) and P2 (yellow) subdomains are indicated. Source: Ossiboff et al., [41]. (TIF) [file pone.0194618.s002.tif]

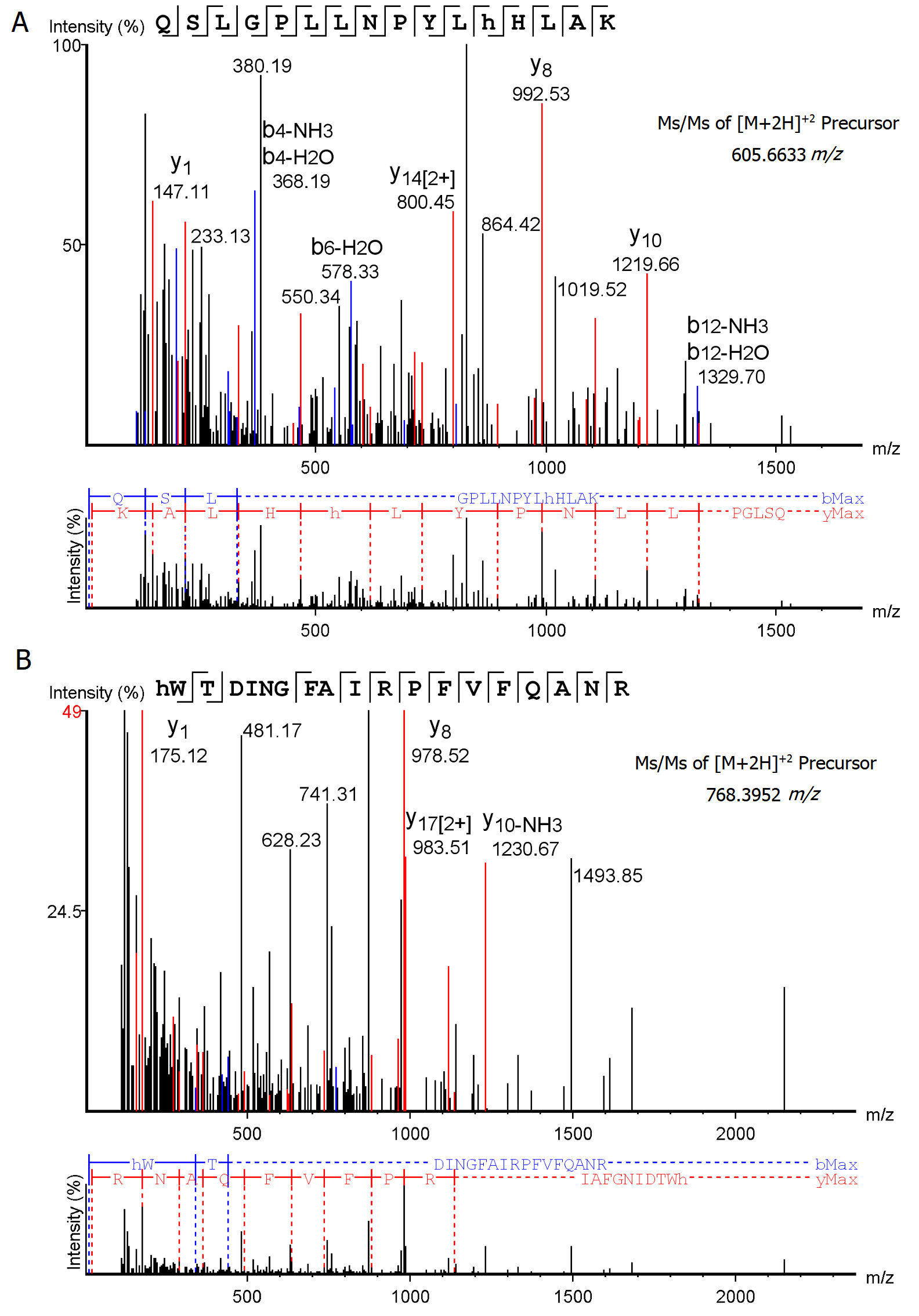

Supplement: S2 Fig — LC-MS/MS annotated spectrum (top) with alignment (bottom) of: A) monoisotopic [M + 2H]2+ observed precursors 605.6633 m/z matched to peptide QSLGPLLNPYLH202HLAK, PEAKS -10logP score 31.22. B) Monoisotopic [M + 2H]2+ observed precursor 768.3952 m/z matched to peptide H354WTDINGFAIRPFVFQANR PEAKS -10logP score 22.87. Theoretical b- and y-type fragment ion types matched to experimental product ion peaks are labeled (spectrum copied and pasted from PEAKS® Studio 7.0). The identified b and y ions are mapped onto the primary sequence and demonstrate modifications in the identified peptides. The peptide scores for the set of representative peptides have a false discovery rate of 0.5%. (TIF) [file pone.0194618.s003.tif]
